# Evidence of an Overweight/Obesity Transition among School-Aged Children and Youth in Sub-Saharan Africa: A Systematic Review

**DOI:** 10.1371/journal.pone.0092846

**Published:** 2014-03-27

**Authors:** Stella K. Muthuri, Claire E. Francis, Lucy-Joy M. Wachira, Allana G. LeBlanc, Margaret Sampson, Vincent O. Onywera, Mark S. Tremblay

**Affiliations:** 1 Children's Hospital of Eastern Ontario Research Institute, Ottawa, Ontario, Canada; 2 University of Ottawa, Ottawa, Ontario, Canada; 3 Kenyatta University, Nairobi, Kenya; McGill University, Canada

## Abstract

**Background:**

Prevalence of childhood overweight/obesity has increased considerably in recent years. The transition to higher rates of overweight/obesity has been well documented in high income countries; however, consistent or representative data from lower income countries is scarce. It is therefore pertinent to assess if rates of overweight/obesity are also increasing in lower income countries, to inform public health efforts.

**Objective:**

This systematic review aimed to investigate the evidence for an overweight/obesity transition occurring in school-aged children and youth in Sub Saharan Africa.

**Methods:**

Studies were identified by searching the MEDLINE, Embase, Africa Index Medicus, Global Health, Geobase, and EPPI-Centre electronic databases. Studies that used subjective or objective metrics to assess body composition in apparently healthy or population-based samples of children and youth aged 5 to 17 years were included.

**Results:**

A total of 283 articles met the inclusion criteria, and of these, 68 were used for quantitative synthesis. The four regions (West, Central, East, and South) of Sub Saharan Africa were well represented, though only 11 (3.9%) studies were nationally representative. Quantitative synthesis revealed a trend towards increasing proportions of overweight/obesity over time in school-aged children in this region, as well as a persistent problem of underweight. Weighted averages of overweight/obesity and obesity for the entire time period captured were 10.6% and 2.5% respectively. Body composition measures were found to be higher in girls than boys, and higher in urban living and higher socioeconomic status children compared to rural populations or those of lower socioeconomic status.

**Conclusions:**

This review provides evidence for an overweight/obesity transition in school-aged children in Sub Saharan Africa. The findings of this review serve to describe the region with respect to the growing concern of childhood overweight/obesity, highlight research gaps, and inform interventions.

**PROSPERO Registration Number:**

CRD42013004399

## Introduction

Worldwide populations are facing “modern” health risks due to increasing prevalence of overweight and obesity (overweight/obesity), physical inactivity, and sedentary behaviours, which are associated with obesogenic environments. This has caused a shift in the major causes of death from “traditional” health risks associated with poverty such as undernutrition, unsafe water, and poor sanitation, to a growing burden of modifiable non-communicable diseases (NCDs) [1]. The World Health Organization (WHO) classifies overweight/obesity as the fifth leading cause of global mortality, and one of the greatest health challenges and determinants for various chronic diseases such as heart disease, hypertension, diabetes, and psychosocial problems, in the 21^st^ century [1], [2]–[6]. This growing population health threat has garnered much attention in view of the declaration and global campaign on the prevention and control of NCDs signed by the United Nations in 2011 [7].

While the health benefits of maintaining healthy body weights and an active lifestyle are well established [6], consumption of calorie-dense foods, declines in habitual physical activity, and increases in sedentary behaviour have been on the rise across developing nations [8]. Traditional practices such as walking long distances, and habitual physical labour have been replaced by motorized transport, and sedentary activities, particularly in urban settings [9]. Furthermore, in many Sub Saharan Africa (SSA) countries, an increased level of body fat is associated with beauty, prosperity, health, and prestige, while in contrast, thinness is perceived to be a sign of ill health or poverty [9]. These factors are now leading to increases in the occurrence of overweight/obesity and related risk factors for NCDs in SSA's children and youth [1], [9].

The health risks associated with overweight/obesity are particularly problematic in children due to the potential for long-term health concerns. A growing body of evidence has shown that overweight/obesity in childhood is significantly associated with increased risk of obesity, physical morbidity, and premature mortality in adulthood [10], [11], [12]. Fortunately, children who are able to attain a normal weight by adolescence have better cardiovascular disease risk factor profiles when compared to those that remain overweight [10]. Childhood is therefore a crucial time to learn basic life skills, including proper nutrition, and how to accumulate sufficient levels of activity in order to attain healthy body weights.

While we must recognise the diversity of populations in SSA, there are certain long-term developmental problems in this region that tend to adversely affect most or all of its countries and peoples [13]. Being the poorest continent in the world, with the highest population growth rate, the concern for an immense double burden of disease due to persistent infectious diseases and modern risks such as an overweight/obesity transition is troubling. The need for population wide interventions to reduce or prevent the adoption of less healthy lifestyles and body weights, particularly for children in SSA has never been greater [9], [14].

The objective of this systematic review was to determine if SSA is indeed undergoing an overweight/obesity transition. Specifically, this review aimed to examine time trends in the proportions of overweight/obesity in school-aged children and youth in SSA, thereby highlighting any research gaps and identifying areas of need for healthy active living interventions.

## Methods

### Study inclusion criteria

Studies were included if they reported using either subjective (e.g., parent or self-report questionnaires) or objective (e.g., directly measured) measures of body composition (weight, height, body mass index, waist/hip circumference, skin-folds, or body image assessment) in children aged 5–17 years. No date or language limits were set, but due to feasibility, only studies presented in either English or French were included. In addition, only studies of populations from SSA countries were included.

### Study exclusion criteria

All published, peer-reviewed studies were eligible for inclusion; however, in order to obtain information on a general population living under ordinary conditions, intervention programs and studies were excluded unless they conducted baseline assessments. Studies done on children with chronic conditions were excluded.

### Search strategy

**Figure 1:**
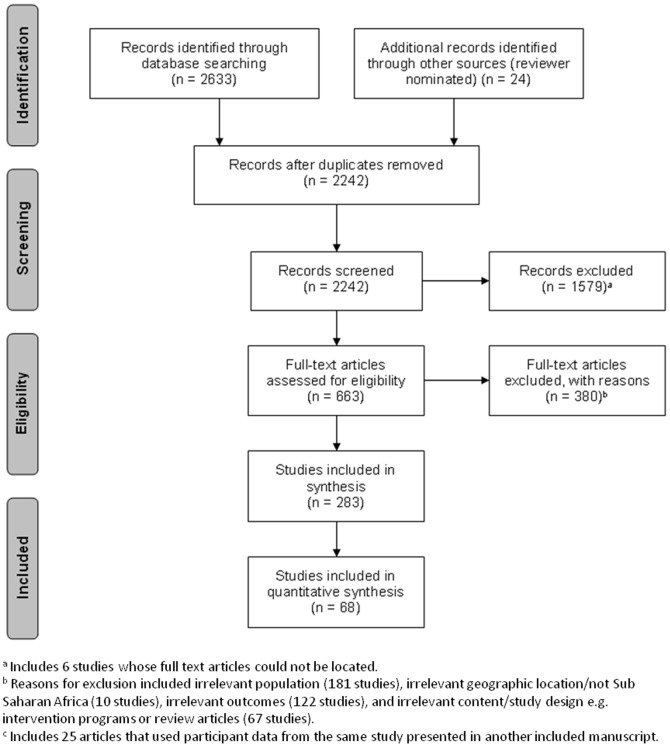
PRISMA flow chart of search strategy results.

Studies were identified using the following electronic databases: Ovid MEDLINE (1948 to May, Week 4, 2013), Ovid Embase (1974 to Week 21, 2013), Africa Index Medicus (database dates not available, latest search on June 3, 2013), Global Health (1973 to June 3, 2013, through the CAB direct interface), Geobase (1884-June 3, 2013 through the Engineering Village interface), and EPPI-Centre database of health promotion research (Bibliomap) (dates of coverage not available, latest search on June 3, 2013). In addition, several open access journals relevant to SSA were identified and those journal web sites were searched for additional relevant papers. The search strategy for this systematic review was completed in tandem with a sister publication examining the evidence for a physical activity, sedentary behaviour, and physical fitness transition among school-age children and youth in SSA; hence, the inclusion of these terms in the search strategy. The search strategy was created and run by MS. The complete search strategy used for MEDLINE is presented in *table 1*. The PRISMA flow chart in *figure 1* accounts for the number of articles included to inform this systematic review. References were exported, de-duplicated and reviewed using Reference Manager Software (Version 11, Thompson Reuters, San Francisco, CA). Titles and abstracts of potentially relevant articles were screened by two independent reviewers (SKM and one of CEF or LJW), and full text copies were obtained for articles meeting initial screening criteria. Full text articles were screened in duplicate for inclusion in the review (SKM and one of CEF or LJW), and any discrepancies were discussed and resolved by the reviewers. This review is registered with the international prospective register of systematic reviews PROSPERO network (registration number: CRD42013004399); available at http://www.crd.york.ac.uk/prospero/.

**Table 1:** MEDLINE search strategy; Ovid interface.

1	exp “Africa South of the Sahara”/
2	(sub-sahar* or east afric* or south afric* or keny* or (south adj3 sahar*)).mp.
3	1 or 2
4	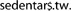
5	Sedentary Lifestyle/
6	((chair or sitting or car or automobile or auto or bus or indoor or in-door or screen or computer) adj time).tw.
7	low energy expenditure.tw.
8	(computer game* or video game* or ((television adj watch*) or tv watch*)).tw.
9	television/or computers/or video games/
10	(screen based entertainment or screen-based entertainment or screen time).tw.
11	physical inactivit*.tw.
12	bed rest.mp.
13	sitting.tw.
14	exp obesity/
15	(obesit* or obese).tw.
16	exp overweight/
17	(overweight or over weight).tw.
18	exp Body Fat Distribution/
19	exp body composition/
20	Waist Circumference/
21	waist circumference.tw.
22	Skinfold Thickness/
23	(skin folds or skin fold*).tw.
24	(body composition* or BMI or body mass index).tw.
25	exp “body weights and measures”/
26	(bio-impedance analysis or BIA).tw.
27	Absorptiometry, Photon/
28	(absorptiometery or densitometry or photodensitometry or DXA or DEXA).tw.
29	Physical Fitness/
30	(physical conditioning or physical fitness).tw.
31	musculoskeletal fitness.tw.
32	physical endurance/
33	cardiovascular fitness.tw.
34	motor 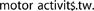
35	physical exertion/
36	aerobic exercise.tw.
37	exp sports/
38	play/
39	exp physical education/
40	musculoskeletal physiological processes/or exercise/or movement/or locomotion/or running/or swimming/or walking/or motor activity/
41	or/4-40
42	(child* or adolescent* or youth* or pediatric* or paediatric*).tw.
43	3 and 41 and 42

**Note:** The search strategy for this systematic review was completed in tandem with a sister publication examining the evidence for a physical activity, sedentary behaviour, and physical fitness transition among school-age children and youth in Sub Saharan Africa; hence, the inclusion of these terms in the search strategy.

### Data extraction, quality assessment, and synthesis

Data extraction was completed using a standardized data extraction template (SKM, CEF, AGL, and LJW). Study quality was assessed by SKM and CEF using a modified Downs and Black instrument [15]. Due to limitations in study design, questions selected from the Downs and Black quality assessment instrument excluded any questions that referred to intervention and trial study methodology. Ten out of the possible 27 questions were used for quality assessment as represented in *table 2*. *Table 3* provides the score out of ten for all studies included in this systematic review. Due to heterogeneity in study methodology and cut-points used to categorize samples into under, healthy, overweight, and obese, we were unable to carry out a meta-analysis in this review. However, quantitative syntheses were conducted by calculating the weighted averages (by sample size) of the prevalence of overweight/obesity. Our goal was to examine time trends and thereafter compute an overall prevalence of overweight/obesity in the region, by comparing the crude rates or prevalence of overweight/obesity in the individual populations or samples. As such, we attempted to standardize the crude rates by acknowledging and adjusting with respect to the sample sizes in each of the included studies, and indicating graphically the sample size upon which a particular data point was based. Findings from the quantitative synthesis were also complemented with narrative syntheses of the included studies.

**Table 2:** Modified Downs and Black checklist (Downs & Black, 1998).

***Reporting***	
**Objective Clearly Stated**	**Question 1 from full checklist (Y = 1/N = 0)**
Main Outcomes Clearly Described	Question 2 (Y = 1/N = 0)
Patient Characteristics Clearly Defined	Question 3 (Y = 1/N = 0)
Main Findings Clearly Defined	Question 6 (Y = 1/N = 0)
Random Variability in Estimates Provided	Question 7 (Y = 1/N = 0)
Actual Probability Values Reported	Question 10 (Y = 1/N = 0)
***External Validity***	
Sample Targeted Representative of Population	Question 11 (Y = 1/N = 0)
Sample Recruited Representative of Population	Question 12 (Y = 1/N = 0)
***Internal Validity/Bias***	
Statistical Tests Used Appropriately	Question 18 (Y = 1/N = 0)
Primary Outcomes Valid/Reliable	Question 20 (Y = 1/N = 0)

**Table 3:** Descriptive characteristics of included studies.

First Author	Year	Study Design	Country	Sample Size	Age Range (Years)	Body Composition Measure or Categorization System	D&B Score
Prinsloo [104]	1964	Cross sectional	South Africa	261	5–6	Weight, height	7
Sloan [105]	1967	Cross sectional	South Africa	393	15–17	Weight, height	7
Smit [106]	1967	Cross sectional	South Africa	2250	6–15	Weight, height, skin fold measures	7
Leary [107]	1969	Cross sectional	South Africa	301	7–15	Weight, height	7
Areskog [108]	1969	Cross sectional	Ethiopia	153	9–14	Weight, height, skin fold measures	7
Fisher [109]	1970	Cross sectional	Zambia	195	7–16	Weight, height	7
Davies [266]	1971	Cross sectional	Rhodesia (now Zimbabwe)	252	7–15	Harvard standards	7
Davies [111]	1973	Cross sectional	Tanzania	141	7–17	Weight, height	7
Davies [110]	1974	Cross sectional	Tanzania	1038	7–16	Weight, height	7
Walker [112]	1974	Cross sectional	South Africa	400	16–17	Weight, height	5
Margo [268]	1976	Cross sectional	South Africa	195	5–16	None OW/OB	7
Booyens [113]	1977	Cross sectional	South Africa	488	6–7	Weight, height	6
Richardson [267]	1977	Cross sectional	South Africa	804	17	Harvard standards	7
Richardson [270]	1977	Cross sectional	South Africa	6598	7–17	None OW/OB	6
Richardson [271]	1977	Cross sectional	South Africa	4655	7, 12 and 17	None OW/OB	7
van Rensburg [272] a	1977	Cross sectional	South Africa	488	6–7	None OW/OB	6
Clegg [114]	1978	Cross sectional	Ethiopia	203	5–16	Weight, height	6
Coovadia [269]	1978	Cross sectional	South Africa	5743	5–12	Weight, height	3
Walker [273]	1978	Cross sectional	South Africa	705	10–12	None OW/OB	7
Sukkar [274] a	1979	Cross sectional	Sudan	855	5–13	Tanner et al., 1966	7
Haller [154]	1980	Cross sectional	Côte d'Ivoire	430	5–15	None OW/OB	6
Walker [115]	1980	Cross sectional	South Africa	1240	16–17	Weight, height	7
Grassivaro [116] b ^,^ c	1980	Cross sectional	Somalia	1206	6–17	Weight, height	9
Rao [117] b	1981	Cross sectional	Zambia	2487	5–17	Weight, height	7
Carswell [275]	1981	Cross sectional	Tanzania	238	10–14	Tanner et al., 1966	6
Singer [118]	1981	Cross sectional	Namibia	306	5–17	Weight, height	7
Oyemade [276]	1981	Cross sectional	Nigeria	353	6–14	None OW/OB	7
Griffin [277]	1982	Cross sectional	Kenya	109	7–13	Other NCHS based system	7
Nnanyelugo [155]	1982	Cross sectional	Nigeria	1347	6–15	Weight, height	7
Kulin [119]	1982	Cross sectional	Kenya	656	10–17	Weight, height	7
Sukkar [278]	1982	Mixed	Sudan	1864	5–14	Harvard standards	7
Power [279] b ^,^ c	1982	Cross sectional	South Africa	790	6–8	Other NCHS based system	9
Richardson [280] b ^,^ c	1983	Cross sectional	South Africa	1337	8, 11, 14, and 17	Harvard standards	9
Akesode [281]	1983	Cross sectional	Nigeria	394	6–17	Other categorization system	7
Little [120]	1983	Cross sectional	Kenya	265	5–17	Weight, height	7
Ng'andu [282]	1984	Cross sectional	Zambia	374	7–14	Other WHO based system	6
Rekart [121]	1985	Cross sectional	Sudan	227	13–17	Weight, height	6
Stephenson [122]	1985	Cross sectional	Kenya	12	7–15	Weight, height, skin fold measures	7
Ndamba [123]	1986	Cross sectional	Zimbabwe	147	8–15	Weight, height	7
Ogunranti [124]	1986	Cross sectional	Nigeria	1165	5–12	Weight	7
Corlett [125]	1986	Cross sectional	Botswana	721	6–14	Weight, height	7
Adams-Campbell [126]	1987	Cross sectional	Nigeria	254	6–17	BMI	7
Wagstaff [243]	1987	Longitudinal	South Africa	864	5–14	NCHS reference	6
Villiers [244]	1987	Cross sectional	South Africa	375	10–17	NCHS reference	7
Ogunranti [127]	1987	Cross sectional	Nigeria	600	5–10	Mid upper arm circumference	5
Corlett [128]	1988	Cross sectional	Botswana	612	7–12	Weight, height	7
Corlett [129]	1988	Cross sectional	Botswana	483	7–14	Weight, height	7
Adeniran [130]	1988	Cross sectional	Nigeria	18	13–17	Weight, height, body fat %	7
Adeniran [131]	1988	Cross sectional	Nigeria	23	13–17	Weight, height, body fat %	7
Jacobs [283]	1988	Cross sectional	South Africa	430	5–10	Other NCHS based system	6
Sigman [16]	1989	Longitudinal	Kenya	138	7 and 8	Weight z-scores	7
Prazuck [132] b ^,^ c	1989	Cross sectional	Mali	844	15–17	Weight, height	9
Ekpo [17]	1990	Cross sectional	Nigeria	1552	5–16	BMI	6
Walker [284] b ^,^ c	1991	Cross sectional	South Africa	1015	14–17	Other NCHS based system	7
Neumann [18]	1992	Cross sectional	Kenya	133	7–9	Weight, height	7
Ng'andu [133]	1992	Cross sectional	Zambia	372	7–16	BMI	7
Benefice [19]	1992	Cross sectional	Senegal	100	9–14	BMI	7
Goduka [134]	1992	Cross sectional	South Africa	300	5–6	Weight, height	7
Adams-Campbell [20]	1992	Longitudinal	Nigeria	208	6–17	Skin fold measures	7
Williams [21]	1992	Cross sectional	Kenya and Nigeria	350	10–15	BMI	6
Ng'andu [285]	1992	Cross sectional	Zambia	800	12–17	Nominal/adjusted classification system	7
Oli [135]	1994	Cross sectional	Ethiopia	1850	7–14	Weight, height	7
McDonald [22]	1994	Longitudinal	Kenya	138	7–8	Weight z-scores	8
Lawless [23]	1994	Longitudinal	Kenya	86	6–11	Weight z-scores	7
Mabrouk [136]	1995	Cross sectional	Sudan	400	7–12	Weight, height	7
Dufetel [137]	1995	Cross sectional	Senegal	72	8–14	Weight, height	7
Walker [156] b ^,^ c	1996	Cross sectional	Nigeria	1192	6–12	None OW/OB	8
Proctor [24]	1996	Cross sectional	Cameroon	119	9–14	BMI	7
Benefice [25]	1996	Cross sectional	Senegal	348	5–13	Weight, height, skin fold measures	7
Pettifor [26]	1997	Cross sectional	South Africa	651	6–17	BMI z-scores	8
Brabin [138]	1997	Cross sectional	Nigeria	914	14–17	Weight, height	7
Cole [286]	1997	Cross sectional	Nigeria	22	11–17	Ketz 1990 system	7
Owa [287]	1997	Cross sectional	Nigeria	904	5–15	US reference sample	8
Longo-Mbenza [27]	1998	Cross sectional	Zaire (now Democratic Republic of Congo - DRC)	4848	5–16	BMI	6
Benefice [157]	1998	Cross sectional	Senegal	348	5–13	None OW/OB	7
Prista [158]	1998	Cross sectional	Mozambique	593	8–15	None OW/OB	8
Benefice [28]	1999	Cross sectional	Senegal	221	12–13	BMI	8
Oelofse [159]	1999	Cross sectional	South Africa	131	5–11	None OW/OB	6
Levitt [29]	1999	Prospective Cohort Study	South Africa	818	5	BMI	7
Monyeki [245]	1999	Cross sectional	South Africa	1149	5–10	NCHS reference	8
Nyirongo [160]	1999	Cross sectional	Zimbabwe	930	5–16	None OW/OB	8
Akinkugbe [139]	1999	Cross sectional	Nigeria	1076	11–15	Weight, height	8
Sellen [30] a	1999	Cross sectional	Tanzania & Kenya	234	5–17	BMI	7
Hamidu [140]	2000	Cross sectional	Nigeria	1712	5–16	Weight, height	7
Sellen [288]	2000	Cross sectional	Tanzania	169	5–12	Seoane & Latham 1971	8
Dibba [141]	2000	Cross sectional	Gambia	160	8–11	Weight, height	8
Zverev [161]	2001	Cross sectional	Malawi	493	6–17	None OW/OB	7
Garnier [31]	2001	Cross sectional	Senegal	80	13–15	BMI	8
Benefice [32] a	2001	Cross sectional	Senegal	40	13	BMI	8
Benefice [33] a	2001	Cross sectional	Senegal	40	13	BMI	8
Jinabhai [246] a	2001	Cross sectional	South Africa	579	8–10	NCHS reference	7
Beasley [162]	2002	Cross sectional	Chad	1024	6–15	None OW/OB	7
Pawloski [34]	2002	Cross sectional	Mali	1056	10–17	Weight z-scores	7
Perzanowski [142]	2002	Cross sectional	Kenya	265	8–15	Weight, height, body fat %	6
Underhay [187] a ^,^ b	2002	Cross sectional	South Africa	1242	10–15	IOTF categories	9
Bhargava [35]	2003	Longitudinal	Kenya	100	6–9	BMI	6
Eckhardt [36]	2003	Cross sectional	South Africa	86	6–16	BMI	7
Garnier [37]	2003	Cross sectional	Senegal	331	14–16	Weight z-scores	8
Grillenberger [38]	2003	Cross sectional	Kenya	110	7	Weight z-scores	7
Mabalia-Babela [289]	2003	Cross sectional	DRC	1087	6–13	BMI percentiles per Rolland-Cachera 1994	8
Mukundi [163]	2003	Cross sectional	Kenya	851	10–17	None OW/OB	7
Prista [247]	2003	Cross sectional	Mozambique	2316	6–17	NCHS reference	8
Leman [39]	2003	Cross sectional	Nigeria	39	5–8	BMI	7
Jinabhai [184] b ^,^ c ^,^ d	2003	Cross sectional	South Africa	29535	8–11	WHO and IOTF categories	9
Schutte [40] a ^,^ b	2003	Cross sectional	South Africa	1244	10–15	BMI	9
Gray [143]	2004	Cross sectional	Kenya	183	5–16	Weight, height	7
Micklesfield [290]	2004	Cross sectional	South Africa	198	7–11	US reference sample	7
Larsen [41]	2004	Cross sectional	Kenya	11	15–17	BMI	7
Benefice [188]	2004	Cross sectional	Senegal	507	16	IOTF categories	6
Benefice [42]	2004	Cross sectional	Senegal	40	13–15	Weight z-scores	7
Monyeki [43]	2004	Longitudinal	South Africa	85	7	BMI	8
McVeigh [44] a	2004	Cross sectional	South Africa	386	9	BMI	7
Cameron [144] a	2004	Cross sectional	South Africa	214	9	Body fat %	7
Mukuddem-Petersen [164] a ^,^ b	2004	Cross sectional	South Africa	1257	10–15	None OW/OB	9
Prista [45]	2005	Cross sectional	Mozambique	2271	16–17	BMI	8
Agyemang [189] b ^,^ c	2005	Cross sectional	Ghana	1277	8–16	IOTF categories	9
Garnier [165]	2005	Longitudinal	Senegal	1806	5–17	CDC categories	7
Calvert [291]	2005	Cross sectional	South Africa	393	8–12	BMI z-score	8
Monyeki [292]	2005	Longitudinal	South Africa	855	7–14	US reference sample	8
Benefice [46]	2005	Cross sectional	Senegal	99	10–13	BMI	8
Friedman [47]	2005	Cross sectional	Kenya	272	10–13	BMI z-score	8
Jinabhai [190] b ^,^ c ^,^ d	2005	Secondary analysis	South Africa	643	8–11	IOTF categories	9
Steyn [248] b ^,^ c ^,^ d	2005	Secondary analysis	South Africa	544	7–8	NCHS reference	10
Underhay [192] a ^,^ b	2005	Cross sectional	South Africa	1242	10–15	IOTF categories	9
Monyeki [191] a	2006	Longitudinal	South Africa	1884	6–13	IOTF categories	8
Zerfu [249]	2006	Cross sectional	Ethiopia	1208	9–17	NCHS reference	6
Armstrong [193] b ^,^ c ^,^ d	2006	Cross sectional	South Africa	10195	6–13	IOTF categories	10
Kruger [194] b	2006	Cross sectional	South Africa	1257	10–15	IOTF categories	9
Aandstad [48]	2006	Cross sectional	Tanzania	156	9–10	BMI	7
Munday [49]	2006	Longitudinal	Gambia	62	5–10	BMI z-scores	7
Djarova [50]	2006	Cross sectional	Zimbabwe	49	6–14	BMI	6
Onyewadume [51]	2006	Cross sectional	Botswana	30	11–14	BMI	8
Nyati [145] a	2006	Cross sectional	South Africa	369	9	Weight, height	8
Vidulich [52] a	2006	Cross sectional	South Africa	476	10	BMI	7
Micklesfield [146]	2007	Cross sectional	South Africa	64	9	Weight, height	7
Micklesfield [53]	2007	Cross sectional	South Africa	400	9	BMI	8
Ben-Bassey [54]	2007	Cross sectional	Nigeria	1504	12–17	BMI	8
Longo-Mbenza [250]	2007	Cross sectional	DRC	1535	5–17	NCHS reference	6
Rohner [166]	2007	Cross sectional	Côte d'Ivoire (Ivory Coast)	281	5–15	None OW/OB	7
Jinabhai [195] b ^,^ c ^,^ d	2007	Cross sectional	South Africa	5322	13–17	IOTF categories	10
Madhavan [167]	2007	Cross sectional	South Africa	117	5–14	None OW/OB	7
Vidulich [55]	2007	Cross sectional	South Africa	476	10	BMI	7
Monyeki [56]	2007	Longitudinal	South Africa	702	7–14	Weight z-score	8
Semproli [196]	2007	Cross sectional	Kenya	1,383	5–17	IOTF categories	7
Andries [57]	2007	Longitudinal	South Africa	702	7–14	Weight z-score	7
Bovet [197] b ^,^ c ^,^ d	2007	Cross sectional	Seychelles	4343	12–15	IOTF categories	9
Goon [147]	2007	Cross sectional	Nigeria	2015		Body fat %	8
Travill [293]	2007	Cross sectional	South Africa	720	8–17	Waterlow et al., 1977	7
Makgae [198] a	2007	Longitudinal	South Africa	1902	6–13	IOTF categories	8
Ejike [58]	2008	Cross sectional	Nigeria	923	10–17	BMI	8
Ekpo [168]	2008	Cross sectional	Nigeria	228	5–15	None OW/OB	8
Anyiam [169] b ^,^ c	2008	Cross sectional	Nigeria	3802	5–13	None OW/OB	10
Nienaber [59]	2008	Cross sectional	South Africa	195	15	BMI	8
Olivieri [170]	2008	Cross sectional	Zimbabwe	982	6–17	None OW/OB	7
Monyeki [199]	2008	Longitudinal	South Africa	1817	7–13	IOTF categories	7
Jeremiah [294]	2008	Cross sectional	Nigeria	144	5–8	Other WHO based system	7
Funke [60]	2008	Cross sectional	Nigeria	315	10–17	BMI	7
Lennox [61]	2008	Cross sectional	South Africa	318	15	BMI	8
Goon [62] a	2008	Cross sectional	Nigeria	2015	9–12	BMI	8
Alaofe [251]	2009	Cross sectional	Benin	180	12–17	NCHS reference	7
Prista [227]	2009	Cross sectional	Mozambique	256	6–16	WHO categories	8
Micklesfield [63]	2009	Cross sectional	South Africa	400	9	BMI	8
Demerath [64]	2009	Secondary analysis	South Africa	196	9	Other NCHS based system	8
Cameron [65]	2009	Secondary analysis	South Africa	227	8–11	BMI	7
Hawley [66]	2009	Secondary analysis	South Africa	1164	9–11	Weight z-scores	6
Berntsen [67]	2009	Cross sectional	Tanzania	190	9–10	BMI	8
Dapi [255]	2009	Cross sectional	Cameroon	581	12–16	CDC categories	7
Ayoola [171]	2009	Cross sectional	Nigeria	349	7–16	None OW/OB	7
Senbanjo [68]	2009	Cross sectional	Nigeria	392	5–14	BMI	8
Padez [228]	2009	Cross sectional	Mozambique	1417	9–17	WHO categories	7
Mulugeta [148]	2009	Cross sectional	Ethiopia	413	10–15	BMI z-scores	7
Naiho [69]	2009	Cross sectional	Nigeria	200	5–10	BMI	6
Adegoke [70]	2009	Cross-sectional	Nigeria	704	6–17	BMI	8
Amuta [172]	2009	Cross sectional	Nigeria	600	6–17	None OW/OB	6
Poopedi [74] b ^,^ c	2009	Cross sectional	South Africa	385	10	BMI	10
Kimani-Murage [252] b ^,^ c	2010	Cross sectional	South Africa	1914	5–14	IOTF categories	9
Bamidele [173]	2010	Cross sectional	Nigeria	139	5–15	Other WHO based system	7
Omigbodun [229]	2010	Cross sectional	Nigeria	1503	10–17	WHO categories	7
Harmse [72]	2010	Cross sectional	South Africa	221	13–17	BMI	7
Senbanjo [256]	2010	Cross sectional	Nigeria	392	5–14	CDC categories	8
Goon [73]	2010	Cross sectional	Nigeria	563	12–17	BMI	7
Mosha [230]	2010	Cross sectional	Tanzania	428	6–12	WHO categories	5
Olumakaiye [174]	2010	Cross sectional	Nigeria	315	10–17	Other NCHS based system	8
Goon [186]	2010	Cross sectional	Nigeria	2015	9–12	CDC and IOTF categories	8
Goon [200]	2010	Cross sectional	Nigeria	219	7–14	IOTF categories	7
Opara [231]	2010	Cross sectional	Nigeria	770	5–14	WHO categories	7
Ejike [253]	2010	Cross sectional	Nigeria	563	10–17	NCHS reference	7
Truter [254]	2010	Cross sectional	South Africa	280	9–13	NCHS reference	7
Ansa [75]	2010	Cross sectional	Nigeria	964	10–17	BMI	8
Bogale [175]	2010	Cross sectional	Ethiopia	100	5	None OW/OB	7
Mulugeta [176]	2010	Cross sectional	Ethiopia	413	10–15	None OW/OB	8
Hawkesworth [295]	2010	Cross sectional	Gambia	171	5–10	BMI	8
Poopedi [71]	2011	Cross sectional	South Africa	385	10	BMI	7
Micklesfield [76]	2011	Cross sectional	South Africa	471	13	BMI	7
Salman [257]	2011	Cross sectional	Sudan	304	6–12	CDC categories	7
Nagwa [232]	2011	Cross sectional	Sudan	1138	10–17	WHO categories	7
Griffiths [77]	2011	Mixed	South Africa	281	9–10	BMI	7
Dabone [233]	2011	Cross sectional	Burkina Faso	649	7–14	WHO categories	7
Henry-Unaeze [78] b ^,^ c	2011	Cross sectional	Nigeria	200	12–17	BMI	9
Hadley [79] b ^,^ c	2011	Cross sectional	Ethiopia	1943	13–17	BMI	8
Odenigbo [258]	2011	Cross sectional	Nigeria	119	6–12	CDC categories	7
Thrandrayen [80] b ^,^ c	2009	Retrospective longitudinal	South Africa	672	10 and 15	BMI z-scores	8
Goon [81]	2012	Cross sectional	South Africa	1136	9–13	BMI	7
Kruger [82] b ^,^ c	2012	Cross sectional	South Africa	582 and 462	7–9	Weight z-scores	6
Semproli [83]	2011	Cross sectional	Kenya	1383	5–17	BMI z-scores	7
Koueta [201]	2011	Cross sectional	Burkina Faso	204	13–16	IOTF categories	7
Stevens [84]	2011	Cross sectional	Ghana	181	9–16	BMI	7
Peltzer [202] d	2011	Secondary analysis	Ghana & Uganda	5613	13–15	IOTF categories	9
Goon [234] a ^,^ b ^,^ c	2011	Cross sectional	Nigeria	2015	9–12	WHO categories	9
Nwizu [85]	2011	Cross sectional	Nigeria	728	10–17	BMI	7
Naude [86]	2011	Cross sectional	South Africa	162	12–16	BMI z-scores	5
Abolarin [87]	2011	Cross sectional	Nigeria	560	6–12	BMI	8
Abrahams [88]	2011	Cross sectional	South Africa	717	10–12	BMI z-scores	8
Motswagole [89]	2011	Cross sectional	South Africa	919	9–15	BMI	8
Croteau [259]	2011	Cross sectional	Kenya	72	8–12	CDC categories	8
Fetuga [226]	2011	Cross sectional	Nigeria	1690	6–16	CDC categories	7
Rankin [149]	2011	Cross sectional	South Africa	81	13–16	Weight	7
Larbi [260]	2011	Cross sectional	Ghana	1482	6–15	CDC categories	8
Mchiza [90]	2011	Secondary analysis	South Africa	201	9–12	BMI	6
Armstrong [203] b ^,^ c ^,^ d	2011	Secondary analysis	South Africa	30365	8–11	IOTF categories	10
Benefice [91]	2011	Cross sectional	Senegal	791	5–15	BMI	8
Kimani-Murage [204]	2011	Cross sectional	South Africa	944	10–14	IOTF categories	7
Dapi [92]	2011	Cross sectional	Cameroon	227	12–16	BMI	8
Vidulich [150]	2011	Cross sectional	South Africa	419	10	Weight, height	8
Faye [296] b ^,^ c	2011	Cross sectional	Senegal	2356	11–17	Rolland-Cachera et al., 1991	8
Fetuga [235]	2011	Cross sectional	Nigeria	1016	6–10	WHO categories	8
Cameron [93]	2011	Cross sectional	South Africa	119	9–10	BMI	6
Goon [151] b	2011	Cross sectional	South Africa	1136	9–13	Weight, height	9
Amusa [205]	2011	Cross sectional	South Africa	409	7–13	IOTF categories	8
Ramos [177]	2011	Cross sectional	Kenya	215	9–10	None OW/OB	7
Puckree [236]	2011	Cross sectional	South Africa	120	10–12	WHO categories	7
Armstrong [94] a ^,^ d	2011	Cross sectional	South Africa	10295	6–13	BMI	10
Adamo [206] a	2011	Cross sectional	Kenya	179	9–13	IOTF categories	7
Goon [261]	2011	Cross sectional	Nigeria	553	12–1 7	CDC categories	7
Kamau [237] b ^,^ c	2011	Cross sectional	Kenya	5325	10–15	WHO categories	9
Ojofeitimi [297]	2011	Cross sectional	Nigeria	280	10–14	Other similar study	8
Kemp [207] b ^,^ c	2011	Cross sectional	South Africa	816	6–7	IOTF categories	10
Oldewage-Theron [238]	2011	Cross sectional	South Africa	97	6–13	WHO categories	7
Okoh [262]	2012	Cross sectional	Nigeria	1302	6–12	CDC categories	7
Naidoo [263]	2012	Cross sectional	South Africa	170	7–10	CDC categories	7
Ene-Obong [209]	2012	Cross sectional	Nigeria	1,599	5–9	IOTF categories	7
Prentice [152]	2012	Longitudinal	Gambia	80	8–16	Weight, height	7
Kramoh [239]	2012	Cross sectional	Côte d'Ivoire	2038	12	WHO categories	7
Musa [95]	2012	Cross sectional	Nigeria	3243	9–15	BMI	7
Adesina [96]	2012	Cross sectional	Nigeria	884	10–17	BMI	8
Cordeiro [178] b ^,^ c	2012	Cross sectional	Tanzania	670	10–15	None OW/OB	9
Monyeki [210] a	2012	Longitudinal	South Africa	256	14	IOTF categories	8
Griffiths [211]	2012	Cross sectional	South Africa	358	16	IOTF categories	7
Onywera [185]	2012	Cross sectional	Kenya	169	9–12	WHO categories	7
Bafor [97]	2012	Cross sectional	Nigeria	369	5–10	BMI	7
Reddy [212] b ^,^ c ^,^ d	2012	Secondary analysis	South Africa	9522 and 9371	14–17	IOTF categories	9
Opare-Addo [240]	2012	Cross sectional	Ghana	720	7–17	WHO categories	8
Ojiambo [98]	2012	Cross sectional	Kenya	200	12–16	BMI z-scores	7
Chinedu [264]	2012	Cross sectional	Nigeria	926	6–16	CDC categories	5
Craig [179]	2012	Cross sectional	South Africa	1519	7, 11, and 15	None OW/OB	5
Amare [180]	2012	Cross sectional	Ethiopia	100	5–15	None OW/OB	8
Moselakgomo [213]	2012	Cross sectional	South Africa	1172	10–16	IOTF categories	8
Micklesfield [214]	2012	Cross sectional	South Africa	381	11–15	IOTF categories	6
Monyeki [215]	2012	Cross sectional	South Africa	256	14	IOTF categories	8
Monyeki [298]	2012	Cross sectional	South Africa	153	14–15	Not indicated	8
Truter [216]	2012	Cross sectional	South Africa	280	9–13	IOTF categories	7
Musa [217]	2012	Cross sectional	Nigeria	3240	9–16	IOTF categories	8
Bovet [218] b ^,^ c	2012	Cross sectional	Seychelles	8462	9–16	IOTF categories	9
Fetuga [299]	2012	Cross sectional	Nigeria	1557	5–11	Weight standard deviation scores	8
Girma [100]	2012	Cross sectional	Ethiopia	116	7–9	Weight z-scores	6
Motswagole [241]	2012	Cross sectional	South Africa	2111	6–15	WHO categories	7
Wolff [101]	2012	Cross sectional	Madagascar	1236	6–15	BMI	8
Toriola [265] a	2012	Cross sectional	South Africa	1172	10–16	CDC categories	7
Wolff [102] a	2012	Cross sectional	Madagascar	1236	6–15	BMI	7
Goon [153] a ^,^ b ^,^ c	2012	Cross sectional	Nigeria	2015	9–12	Weight, height	10
Toriola [219]	2012	Longitudinal	South Africa	283	14	IOTF categories	8
Feeley [208]	2013	Longitudinal	South Africa	1298	13, 15, and 17	IOTF categories	7
Wilson [220] b ^,^ c ^,^ d	2013	Secondary analysis	Seychelles	580	11–17	IOTF categories	8
Ginsburg [221]	2013	Cross sectional	South Africa	1613	15	IOTF categories	7
Senbanjo [103]	2013	Cross sectional	Nigeria	548	5–17	BMI	7
Malete [222]	2013	Cross sectional	Botswana	756	13–16	IOTF categories	7
Neumann [181]	2013	Cross sectional	Kenya	910	6–14	None OW/OB	7
Degarege [182]	2013	Cross sectional	Ethiopia	403	5–15	None OW/OB	8
Puoane [242]	2013	Cross sectional	South Africa	162	10–15	WHO categories	7
Amare [183]	2013	Cross sectional	Ethiopia	405	9–14	None OW/OB	8
Mang'eni [223] a	2013	Cross sectional	Kenya	200	13–16	IOTF categories	7
Onywera [224] a	2013	Cross sectional	Kenya	179	9–13	IOTF categories	7
Heroux [225] a	2013	Cross sectional	Kenya	179	9–13	IOTF categories	7
						**Average (D&B) score**	**7.4**

**Acronyms:** D & B score (Downs & Black score); None OW/OB (none were overweight/obese); BMI (Body Mass Index); CDC-NCHS (Centers for Disease Control and Prevention – National Center for Health Statistics); WHO (World Health Organization); IOTF (International Obesity Task Force).

a =  Identical study sample as used in another included manuscript (not included in quantitative synthesis) [*n* = 27].

b =  Article indicated targeting a sample size representative of the population of interest [*n* = 38].

c =  Article indicated recruiting a sample size representative of the population of interest [*n* = 31].

d =  Article indicated that the sample size was nationally representative [*n* = 11].

## Results

*Figure 1* shows the PRISMA flow chart with numbers of included and excluded articles at each step of the review process, while *table 3* provides a summary of all studies that met the inclusion criteria. A total of 2657 records were identified through database searches and other sources. Following de-duplication, 2242 articles were screened for eligibility, and 663 articles were selected for a full-text review. Of these, 283 articles met the inclusion criteria, and 68 of the studies (comprising 190,149 participants) were used in quantitative synthesis. Reasons for exclusion included: ineligible population (e.g., studies that did not involve children 5–17 years of age with no pre-existing condition) (n = 181); ineligible country (e.g., population living in a country/region outside of SSA) (10); ineligible outcome (n = 122); or ineligible study design (n = 67). It is important to note that all the studies included in this review were found to have used objective methods of collecting body composition data.

### Regional representation

As shown in *table 3*, which includes a summary of the 283 studies included in the review, the four regions of SSA were well represented, with 91 (32.1%) from West African countries - with Nigeria represented in 60 of these records; 7 (2.5%) from Central African countries; 75 (26.5%) from East African countries - with Kenya represented in 28 of these records; 108 (38.2%) from South African countries - with South Africa represented in 102 of these records; and 2 (0.7%) that were East and West combined. In total, 27 countries were captured in this review.

### Publication rate

The earliest relevant record captured was published in 1964. There was a marked increase in the publishing rate from the earliest to the current studies: 5 articles between 1960 and 1969, 15 from 1970–1979, 32 from 1980–1989, 31 from 1990–1999, 92 from 2000–2009, and 108 articles from 2010 to May/June 2013.

### Data quality assessment

The average modified Downs and Black score out of ten for all studies included in this systematic review was 7.4; indicative that data quality was fairly high among the included records, within the prescribed limitations of study designs included in this review. The majority of studies used in the quantitative synthesis scored 7 or higher. As presented in *table 3*, the scoring process further revealed that only 38 (13.4%) of 283 included articles *targeted* a sample that was representative of their population of interest, and 31 (11.0%) *recruited* a sample that was representative of their population of interest. Only 11 (3.9%) articles explicitly mentioned using a nationally representative sample, one of which used the same study sample as that of another already included study.

### Body composition measures

Of the 283 included studies, 88 (31.1%) articles [16]–[103] reported on mean BMI, BMI-z-score, and/or weight z-scores of the sample population, 50 (17.7%) articles [104]–[153] reported on body fat percentage, waist circumference, skin fold measures, and/or weight and height measures, and a total of 30 (10.6%) articles [154]–[183] reported finding no prevalence of overweight/obesity in their study samples. Of the remaining 115 (40.6%) records, 82 articles [184]–[265] used the more widely accepted international cut-points (namely, the International Obesity Task Force (IOTF), the Centers for Disease Control and Prevention (CDC), and the most recent WHO cut-points) to further categorize their samples into underweight, normal-weight, and overweight/obese. The other 33 articles [266]–[298] mentioned using one of a number of other cut-points and reference standard groups including but not limited to Tanner et al., 1966, Seoane & Latham, 1971, Frisancho 1990, Rosner et al., 1998, Harvard Standards, Waterlow 1972/77, and various US and UK reference samples. Of the 30 studies reporting no prevalence of overweight/obesity, a majority had not used the more widely accepted international cut-points, while the reminder did not provide the required prevalence estimates to be included in the quantitative synthesis.

### Quantitative synthesis

Of the 82 articles that used more widely accepted international cut-points, 11 studies [187], [191], [192], [198], [206], [215], [224], [225], [234], [246], [265] were removed due to having an identical study sample as an already included study, and 3 studies [214], [218], [264] were removed for having not indicated the sample sizes in the age range of interest. As represented in *table 4*, the remaining 68 (24.0%) articles [184]–[186], [188]–[190], [193]–[197], [199]–[205], [207]–[213], [216], [217], [219]–[223], [226]–[233], [235]–[245], [247]–[263] were used in quantitative synthesis. Of these, the largest proportion (44.1%) used the IOTF cut-points [299], 30.9% used CDC cut-points [300], and 25.0% used the most recent WHO cut-points [301] for weight status. Briefly, the IOTF methodology involved obtaining the body mass index for children from six large nationally representative cross sectional surveys on growth from Brazil, Great Britain, Hong Kong, the Netherlands, Singapore, and the United States. Thereafter, centile curves for body mass index were constructed for each dataset by sex, and passed though the widely used cut off points of 25 and 30 kg/m2 for adult overweight and obesity at age 18 years. The resulting curves were averaged to provide age and sex specific cut off points for children 2–18 years of age [299]. In the case of the CDC cut-points, growth charts were developed based on data from five national health examination surveys conducted in the United States, including limited supplemental data. Smoothed percentile curves were created by first smoothing selected empirical percentiles, then creating parameters obtain the final curves, additional percentiles, and z-scores [300]. Finally, the WHO cut-points were developed after data from the 1977 National Center for Health Statistics (NCHS)/WHO growth reference for 1–24 years, were merged with data from the under-fives growth standards' cross-sectional sample to smooth the transition between the two samples. The new curves filled the gap in growth curves and provided an appropriate reference for the 5 to 19 years age group [301].

**Table 4:** Proportions of overweight/obesity as reported by studies used in quantitative synthesis.

					Sample Size (*n*)			Proportions in Males				Proportions in Females				Proportions in Both Males and Females			
First Author	Year	Country	Cut Off	Age Range (years)	M	F	Total	UW	NW	OW	OB	UW	NW	OW	OB	UW	NW	OW	OB
Villiers [244]	1987	South Africa	1	14–15		57	57								1.8				1.8
Wagstaff [243]	1987^a^ (1981)	South Africa	1	5–14			937									27.3		3.9	3.4
Wagstaff [243]	1987^a^ (1983)	South Africa	1	5–14			864									21.9		7.1	4.0
Monyeki [245]	1999	South Africa	1	5–10	595	557	1152			0.5				0.7				0.6	
Prista [247]	2003	Mozambique	1	6–17	1094	1222	2316	21.9		4.8		10.0		7.7		15.6		6.3	
Jinabhai [184]	2003	South Africa	2	8–11	17351	12025	29376			3.0	0.7			4.5	1.2			3.6	0.9
Benefice [188]	2004	Senegal	3	16	188	319	507	50.0		0.0	0.0	17.9		1.6	0.0	29.8		1.0	0.0
Agyemang [189]	2005	Ghana	3	8–16	616	661	1277			3.1				6.4				4.8	
Jinabhai [190]	2005	South Africa	3	8–11	292	351	643											5.1	0.6
Steyn [248]	2005	South Africa	1	7–8			544									6.4	85.4	5.0	3.3
Zerfu [249]	2006	Ethiopia	1	9–17			918									23.8		3.5	
Armstrong [193]	2006	South Africa	3	6–13	5603	4680	10283			10.8	3.2			13.0	4.9			11.8	4.0
Kruger [194]	2006	South Africa	3	10–15	608	649	1257			4.1	1.5			8.3	1.7			6.3	1.6
Longo-Mbenza [250]	2007	DRC	1	≥12	362	124	486			24.0				68.5				35.5	
Jinabhai [195]	2007	South Africa	3	13–17	2398	2924	5322	18.4		4.2		2.6		20.9		9.7		13.4	
Semproli [196]	2007	Kenya	3	5–17	702	681	1383	10.6				6.3						3.2	
Bovet [197]	2007	Seychelles	3	12–15	2202	2141	4343			8.1	3.1			13.1	4.4			10.6	3.7
Monyeki [199]	2008	South Africa	3	7–13	938	879	1817			1.1				2.1				1.6	
Alaofe [251]	2009	Benin	1	12–17		180	180					8.0	81.0	8.0	3.0	8.0	81.0	8.0	3.0
Prista [227]	2009	Mozambique	2	6–16	139	117	256											1.1	0.0
Dapi [255]	2009	Cameroon	1	12–16	248	333	581	6.0		4.0		1.0		14.0		3.0		10.0	
Padez [228]	2009	Mozambique	2	9–17	298	400	698			0.7	0.0			1.3	0.3			1.0	0.2
Goon [186]	2010	Nigeria	1	9–12	979	1036	2015	87.1		2.1	1.6			3.2	2.8			2.7	2.2
Kimani-Murage [252]	2010	South Africa	3	5–14			1884									6.5		5.0	1.5
Omigbodun [229]	2010	Nigeria	2	10–17	763	740	1503	22.3		1.2		15.5		3.9		19.0		2.5	
Senbanjo [256]	2010	Nigeria	1	5–14	202	190	392	37.1	61.9	1.0		23.2	74.7	2.1		30.4	68.1	1.5	
Mosha [230]	2010	Tanzania	2	6–9	60	145	205									21.4	68.8	5.8	4.0
Goon [200]	2010	Nigeria	3	7–14	107	112	219											2.7	1.0
Odenigbo [258]	2010	Nigeria	1	6–12			119									29.4	63.0	6.7	0.8
Opara [231]	2010	Nigeria	2	5–12.5			378									29.1			10.3
Ejike [253]	2010	Nigeria	1	10–17	337	226	563	5.3		23.7		2.7		7.2		4.3		17.1	
Salman [257]	2010	Sudan	1	6–12	68	236	304		82.4	11.8	5.9		75.0	14.0	11.0		76.7	13.5	9.9
Truter [254]	2010	South Africa	1	9–12	128	152	280		78.9	15.6	5.5		77.6	15.1	7.2		78.2	15.4	6.4
Nagwa [232]	2011	Sudan	2	10–17	526	612	1138	17.7	61.0	9.9	11.4	10.6	69.6	11.6	8.2	13.9	65.6	10.8	9.7
Dabone [233]	2011	Burkina Faso	2	7–14	312	337	649									13.7		2.3	
Koueta [201]	2011	Burkina Faso	3	13–16			204											3.9	
Peltzer [202]	2011	Ghana & Uganda	3	13–15	2738	2875	5613			2.7	0.5			9.5	0.9			6.2	0.7
Croteau [259]	2011	Kenya	1	8–12	29	43	72									11.1	84.7	4.2	
Fetuga [226]	2011	Nigeria	2	6–16	821	669	1690			2.5				3.3				2.5	
Larbi [290]	2011	Ghana	1	6–15	706	776	1482									7.9	78.7	13.4	
Kimani-Murage [204]	2011	South Africa	3	10–14			944											7.5	
Fetuga [235]	2011	Nigeria	2	6–10	479	537	1016	23.8		3.8		20.8		3.3		22.2		3.5	
Amusa [205]	2011	South Africa	3	7–13	193	216	409			2.6				2.9				2.8	
Puckree [236]	2011	South Africa	2	10–12	48	72	120									66.2	28.8	5.0	
Goon [261]	2011	Nigeria	1	12–17	0	553	553					5.4	77.0	11.1	5.4	5.4	77.0	11.1	5.4
Kamau [237]	2011	Kenya	2	10–15	2620	2705	5325			6.5	2.6			10.9	3.6			8.7	3.1
Kemp [207]	2011	South Africa	3	6–7	419	397	816		90.2	6.4	3.3		86.4	9.3	4.3		88.4	7.8	3.8
Oldewage-Theron [238]	2011	South Africa	2	6–13	43	54	97	4.7	90.7	2.3	2.3	5.7	90.5	3.8	0.0	5.2	90.7	3.1	1.0
Armstrong [203]	2011^a^ (1994)	South Africa	3	8–11	17756	12609	30365			1.1				1.4				1.2	0.2
Armstrong [203]	2011^a^ (2004)	South Africa	3	8–11	17756	12609	30365			9.5	2.2			16.5	4.4			12.4	3.1
Okoh [262]	2012	Nigeria	1	6–12	585	717	1302									11.7	76.7	5.7	5.9
Naidoo [263]	2012	South Africa	1	7–10	70	100	170		54.3	11.4	34.3		55.0	16.0	29.0		54.7	14.1	31.2
Ene-Obong [209]	2012	Nigeria	3	5–9			706									19.0	68.7	9.5	2.8
Kramoh [239]	2012	Côte d'Ivoire	2	12	856	1182	2038				1.8				6.8	64.0	27.0	4.0	5.0
Monyeki [210]	2012	South Africa	3	14	100	156	256	44.0	48.0	8.0		30.7	51.9	17.3		35.9	50.4	13.7	
Griffiths [211]	2012	South Africa	3	16	190	168	358			6.3	3.7			22.2	8.4			13.3	5.7
Onywera [185]	2012	Kenya	2	9–12	85	84	169			6.8				16.7				12.0	
Opare-Addo [240]	2012	Ghana	2	7–17	0	720	720									6.0	74.6	10.4	8.9
Moselakgomo [213]	2012	South Africa	3	10–16	541	631	1172	4.6	80.8	9.1	5.5	5.2	79.4	11.0	4.4	4.9	80.0	10.1	4.9
Truter [216]	2012	South Africa	3	9–13	128	152	280			15.6	5.5			15.1	7.2			15.5	6.5
Musa [217]	2012	Nigeria	3	9–16	1526	1,714	3240										88.5	9.7	1.8
Motswagole [241]	2012	South Africa	2	6–15			2111									34.2		0.6	
Toriola [219]	2012	South Africa	3	14	111	172	283	34.2	48.6	17.1		26.2	41.0	32.4		29.3	44.0	26.4	
Reddy [212]	2012^a^ (2002)	South Africa	3	14–17	4184	5338	9522			6.3	1.6			24.3	5.0			16.4	3.5
Reddy [212]	2012^a^ (2008)	South Africa	3	14–17	4565	4806	9371			11.0	3.3			29.0	7.5			20.2	5.5
Feeley [208]	2013	South Africa	3	13–17	607	616	1223			8.1				27.0				17.6	
Wilson [220]	2013	Seychelles	3	11–17	278	302	580									13.4		15.6	7.7
Ginsburg [221]	2013	South Africa	3	15	773	840	1613	20.3	71.8	5.4	2.5	9.6	65.4	17.5	7.5	14.2	68.5	11.7	5.1
Malete [222]	2013	Botswana	3	13–16	464	292	756									5.0	78.4	11.6	5.1
Puoane [242]	2013	South Africa	2	10–15	0	162	162									2.4	61.4	36.2	
Mang'eni [223]	2013	Kenya	3	13–16	98	102	200											5.0	
					**95885**	**84455**	**190149**	**25.0**	**68.0**	**5.6**	**2.0**	**8.3**	**68.6**	**11.5**	**3.9**	**17.6**	**68.5**	**8.1**	**2.5**
					**Sample totals**			**(M) – weighted averages**				**(F) – weighted averages**				**(T) – weighted averages**			

**Acronyms:** M (male); F (female); UW (underweight); NW (normal weight); OW (overweight); OB (obese).

**Weighted averages:** Proportions may not add up to 100% for M, F, and T since some of the included studies did not report in each of the UW, NW, OW, and, OB categories.

a Year of publication (year that corresponding data was collected included in brackets).

**Figure 2:**
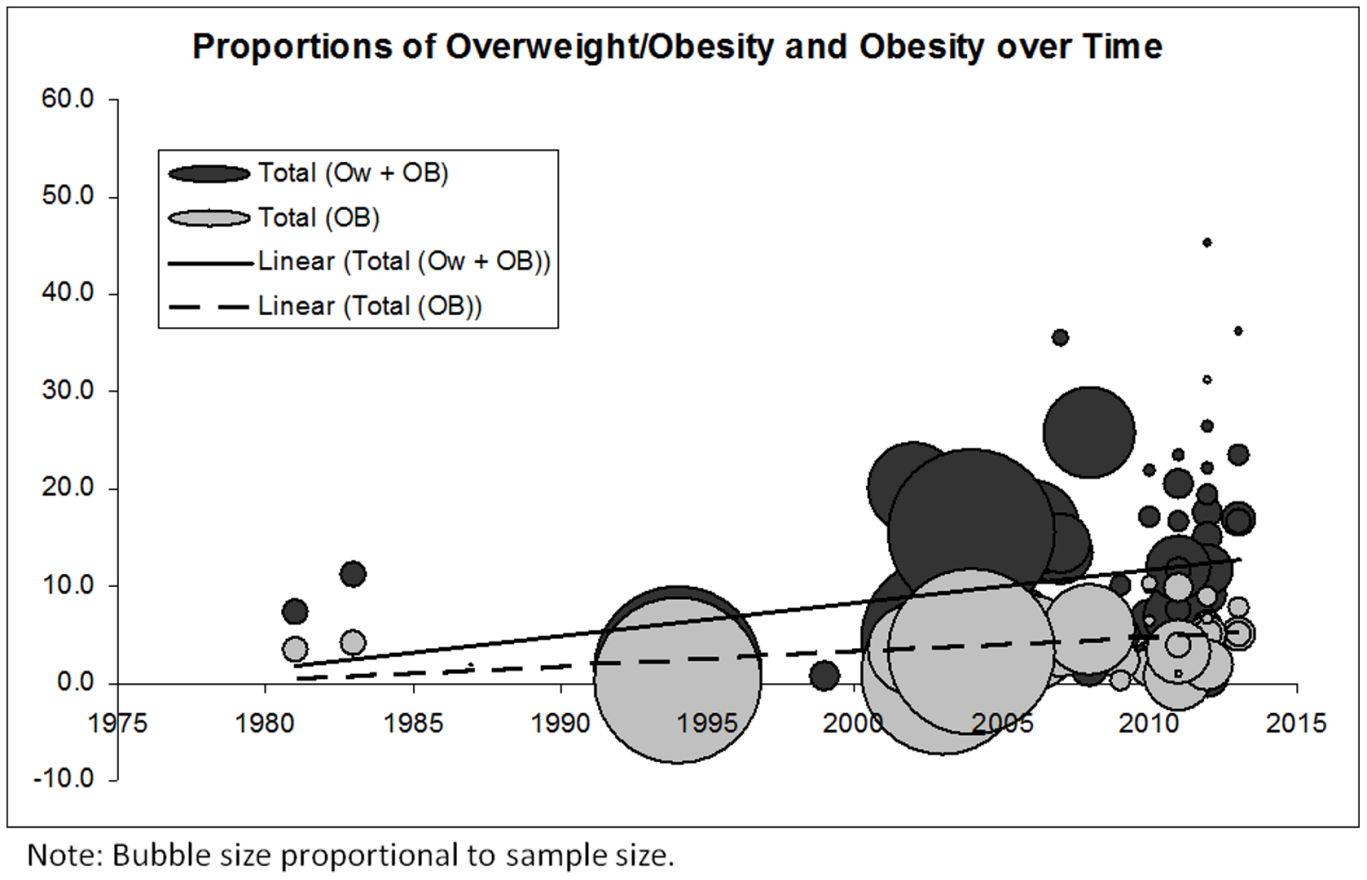
Proportions of overweight/obesity (combined) and obesity over time in Sub Saharan Africa.

**Figure 3:**
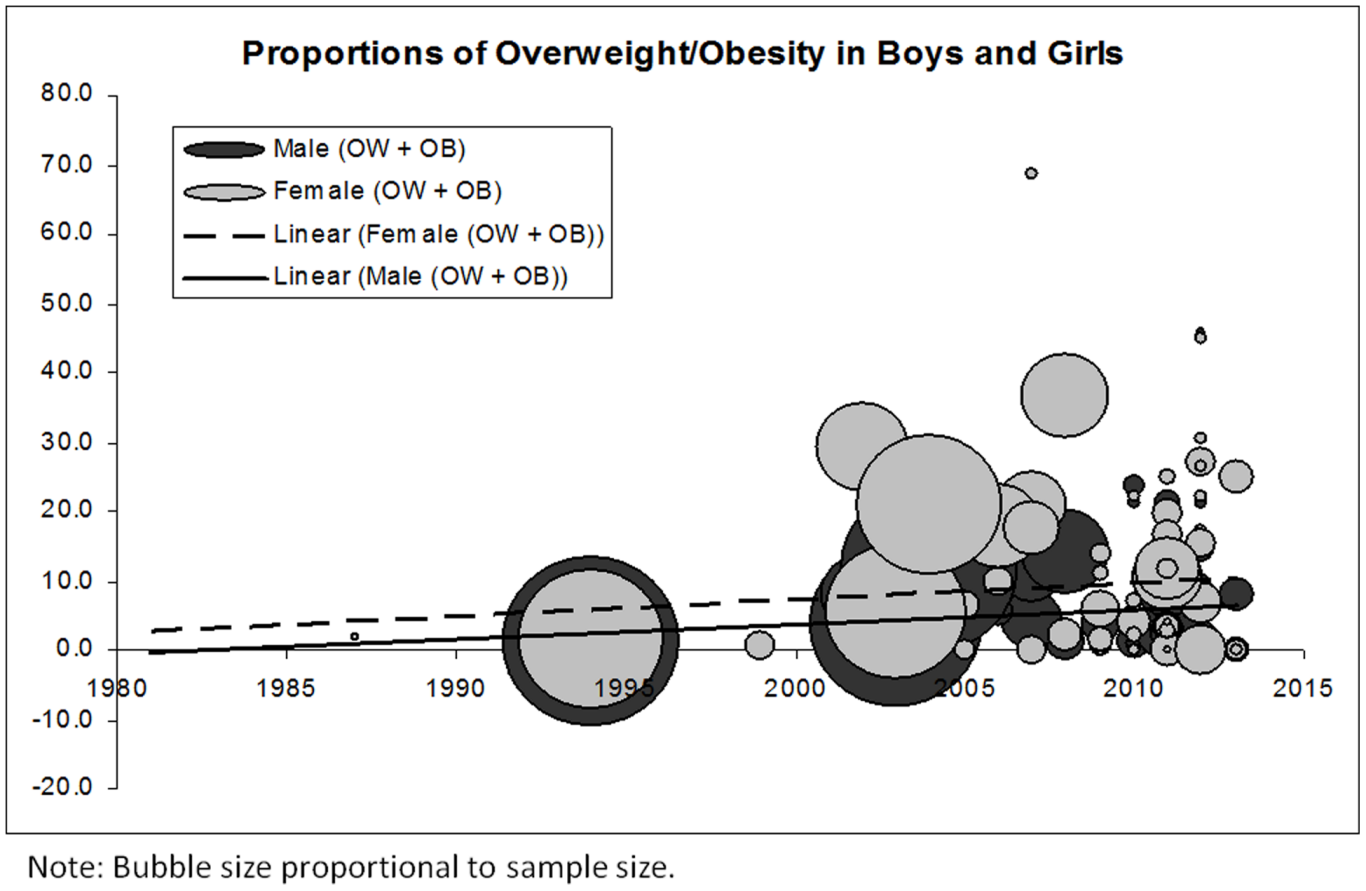
Proportions of overweight/obesity (combined) in Sub Saharan Africa's boys and girls.

**Figure 4:**
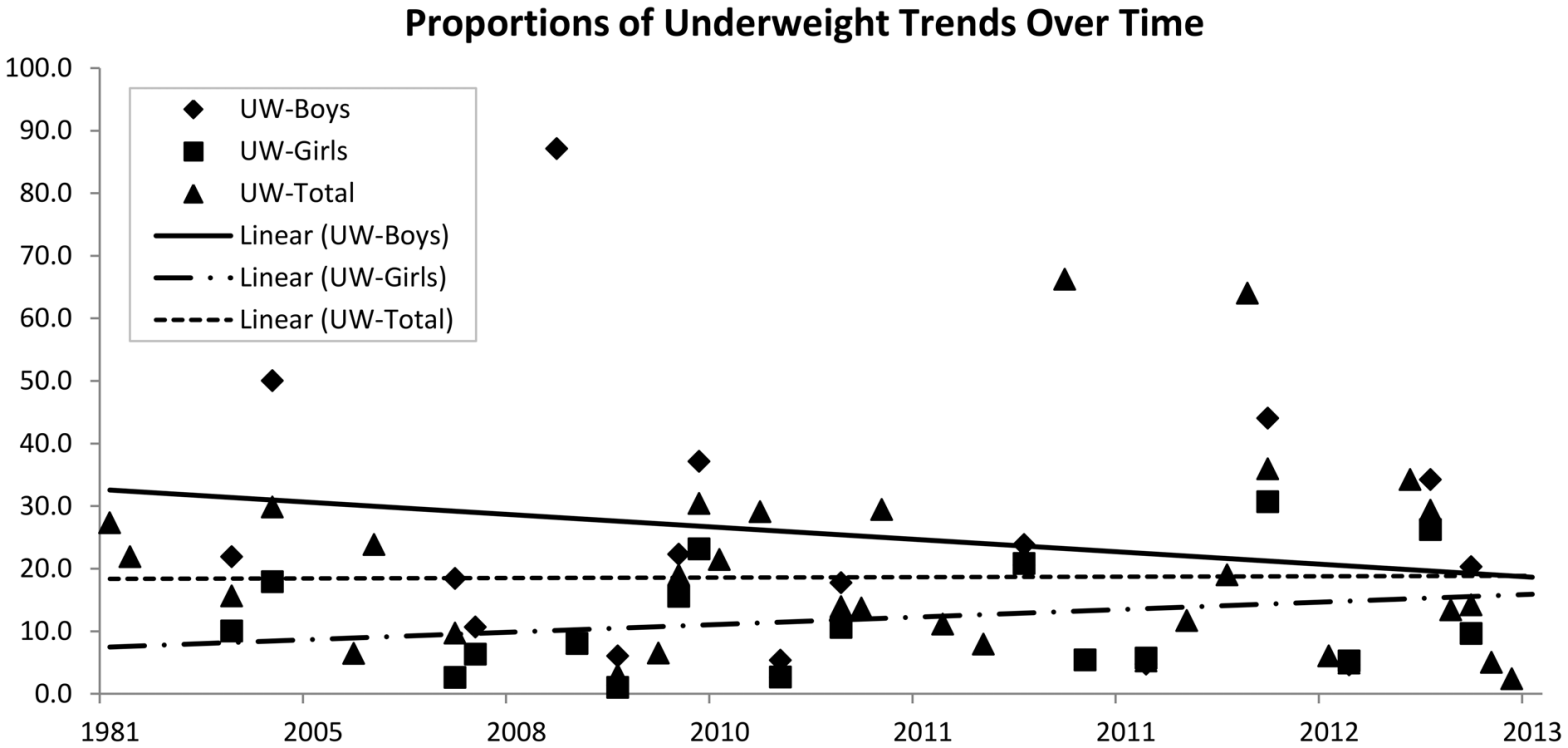
Proportions of underweight over time in Sub Saharan Africa.

*Figure 2* shows a distinctive time trend towards increasing proportions of overweight/obesity in school-aged children in SSA. The figure also shows a similar but less prominent trend towards increasing proportions of obesity over time. *Figure 3* *,* shows increasing trends in proportions of overweight/obesity over time for both boys and girls; however, the proportions are consistently higher in girls than in boys. To determine the robustness of these findings, we examined the trends in overweight/obesity over time using the few studies that indicated having recruited a representative sample of the population. We similarly found a trend towards increasing proportions of overweight/obesity among school-aged children in this region. The findings were also similar when boys and girls were assessed separately. While not the focus of this manuscript, as shown in *Figure 4*, we also examined trends in underweight over time for the included studies that had also reported this proportion. We found a trend towards decreasing proportions of underweight over time in boys, a trend towards increasing proportions over time in girls, and a largely unaltered trend over time - at approximately 20% - when boys and girls were considered together.

The weighted averages (for the entire time period and all studies included in the quantitative analysis) of overweight/obesity proportions in boys and girls were calculated as 7.6% and 15.4% respectively. Weighted averages of obesity alone for boys and girls were 2.0% and 3.9% respectively. Weighted averages of overweight/obesity and obesity proportions for boys and girls combined were 10.6% and 2.5%. Weighted proportion of underweight was calculated as 25.0% for boys, 8.3% for girls, and 17.6% for boys and girls combined.

### Narrative synthesis

Narrative descriptions of the relationship between body composition and age, sex, socioeconomic status (SES), and urban/rural differences are discussed below based largely on the studies not included in the quantitative synthesis:

#### Sex differences

Of the 96 studies [16]–[18], [20], [24]–[26], [29], [36], [39], [40], [43], [45], [46], [48], [51], [53]–[56], [59]–[61], [63]–[68], [70]–[72], [74], [76]–[80], [83], [85], [86], [89], [91], [92], [95], [96], [98], [103]–[105], [107], [109]–[114], [116]–[120], [123], [124], [126], [128], [129], [132], [133], [135], [136], [140], [143], [147], [149]–[152], [163], [169], [170], [214], [215], [218], [225], [281], [287], [289], [292], [293], [295] that reported their data by sex, 31 articles [20], [25], [29], [40], [45], [59], [67], [68], [70], [71], [74], [76], [78], [79], [85], [86], [89], [92], [95], [96], [103], [107], [124], [126], [132], [147], [151], [163], [170], [214], [215] reported that girls had higher body composition measures than boys, while 5 articles [265], [267], [289], [292], [293] reported that boys had higher body composition measures than girls. The remaining studies either found no significant difference or did not report a difference between boys and girls.

#### Urban/rural differences

Thirty-three articles compared body composition measures in urban and rural populations. Of these, 29 studies (including 7 studies used in the quantitative synthesis) [17, 24, 27, 31, 34, 37, 54, 58, 79, 84, 87, 98, 119, 128, 129, 138, 156, 163, 206, 212, 282, 298, (185, 189, 200, 217, 233, 240, 260)] reported significantly higher body composition measures in the urban compared to the rural sample, with the remaining studies [110], [111], [140], [280] reporting no significant difference between the two populations.

#### Socioeconomic status (SES) differences

Twenty four articles reported on outcomes of interest by some measure of socioeconomic status (e.g., income quartile, public/private school attendance). Of these, 19 articles (including 8 studies used in the quantitative synthesis) [45, 54, 61, 68, 75, 77, 84, 92, 99, 101, 156, 163, 169, 218, 296, 297, (212, 228, 231, 237, 247, 250, 255, 256)] reported that higher SES was associated with higher body composition measures, whilst the remaining articles [54], [75], [92], [169], [256] found no significant association of SES on body composition.

#### Age differences

Of the articles that reported on body composition measures by age, 15 studies found a largely positive relationship with age [287], [170], [70], [147], [103], [95], [151], [20], [42], [242], [256], [229], [230], [199], [297], while 7 studies found a largely negative relationship with age [83], [264], [233], [19], [190], [196], [245]. In some cases, the relationship between age and body composition measures differed between sexes; as such, we may conclude that there was no convincing or consistent evidence of an independent age effect.

## Discussion

To our knowledge, this systematic review is the first to comprehensively examine if there is evidence supporting an overweight/obesity transition in school-aged children and youth in SSA.

### An overweight/obesity transition

Due to vast heterogeneity in types of measurement, classification, and analysis, both narrative and quantitative analyses (weighted proportions and bubble plots of overweight/obesity) were presented in this review. Quantitative synthesis was completed using 68 studies that categorized children and youth based on internationally accepted cut-points for weight status. The weighted averages of overweight/obesity proportions in boys and girls was 7.6% and 15.4% respectively, while obesity proportions in boys and girls was 2.0% and 3.9% respectively. Weighted averages of overweight/obesity, and obesity for the total population were 10.6% and 2.5%. Current evidence revealed a clear transition of increasing proportions of overweight/obesity in school-aged children in SSA, and a similar, but less prominent trend towards increasing proportions of obesity over time. This transition to higher proportions of overweight/obesity is similar to observed trends in developed countries; however, the weighted averages fall far below proportions in various high income countries. For example, in Canada, research has shown that the prevalence of overweight/obesity has more than doubled (14% to 29%) and the obesity rate has tripled (3% to 9%) over the last 25 years in children and youth 5 to 17 years of age [302], [303]. In the USA, 33% of children and youth 6–19 years are considered to be overweight/obesity, and 18% are considered to obese [304].

It is important to note that across all age groups, WHO cut-points yield higher proportions of boys and girls classified as overweight/obesity than do the IOTF, or CDC cut-points [305]. While studies that used any of the three cut-points were analysed together in this review, when interpreting prevalence estimates of overweight/obesity, it is important to consider the choice of cut-point used in each study. With the largest proportion of included studies using IOTF cut-points, it could be argued that this may “dilute” the weighted average of the proportions of overweight/obesity calculated for SSA. Nonetheless, these results indicate that while there is an imminent threat of continued increases in levels of childhood overweight/obesity in SSA, implementing viable population health interventions may mitigate the associated health risks in these earlier stages.

### Persistence of underweight

In discussing an overweight/obesity transition, it is important to recognize that child under-nutrition remains one of SSA's most fundamental challenge for improved human development [306], [307], [308]. This is particularly concerning when considering the school-aged child population as malnutrition affects their education outcomes, and consequently opportunities for success in later years [306]. Inadequate access to food and health services as a result of poverty and broader social determinants of health are some of the underlying determinants of child under-nutrition. The underweight trend over time was largely unaltered at approximately 20% for boys and girls combined, providing the evidence of a persisting underweight problem among SSA's children and youth, and substantiating the emergence of a public health double-edged sword. This persistence in underweight coupled with an overweight/obesity transition may place undue strain on the limited healthcare resources in SSA countries [14]. As such, frameworks for interventions to improve the nutritional status of SSA children will have to account for broader concepts such as societal organization, economic structures, and political ideologies [306]. We would however like to caution the reader that describing an underweight trend was not an objective of this systematic review; as such, pertinent articles reporting on underweight may have been omitted during the literature search thereby skewing these results.

### Sex differences

Both quantitative and narrative synthesis revealed that there were increasing trends in proportions of overweight/obesity over time for both boys and girls; however, body composition measures and the proportions of overweight/obesity were proportionally higher in girls than in boys. In contrast, in North America, obesity is more common in boys than in girls, with the most significant differences observed among younger children 5–11 years [304], [309]. Higher proportions of overweight/obesity in SSA girls may be related to differences in gender roles particularly those requiring higher physical exertion (e.g., boys participating in higher energy expending roles/activities); and, cultural desirability whereby being overweight (i.e., “rounder”) is an admired trait and seen as a sign of wealth and prestige, particularly in girls.

### Urban/rural and SES differences

Narrative synthesis revealed higher body composition measures in the urban compared to the rural population. In addition, higher SES was associated with higher body composition measures, pointing to a positive SES relationship. Factors associated with overweight/obesity span various behavioural, social, environmental, and biological constructs making them difficult to ascertain; however, urban residence and higher SES may be positively associated with overweight/obesity in SSA owing to improved access to governance, health care, education, employment and income, in addition to increased availability of packaged foods high in saturated fats and sugars and increased sedentary behaviour, all of which are more accessible to and/or affordable for those of higher SES or individuals living in urban areas.

### Strengths, limitations, and future directions

The main strength of this review was the use of high quality standards to conceptualize and conduct the methodology and synthesis. Further, as many decisions as possible were made *a priori* to limit possible bias, and all levels of the review process were conducted in duplicate, ensuring a higher level of accuracy. Our assessment indicated that the quality of included studies was relatively high. The main limitation of this study lies in the vast heterogeneity in study methodology. The variety in the types of body composition measurements, analyses, definitions of SES, and reference standards limited our interpretation and presentation of the results. Quantitative synthesis was limited to those using the more widely accepted cut-points to further categorise study samples by weight status. It is also unclear if any material relevant for this review may have been published in un-indexed journals and hence not captured by the literature search.

Recognizing that future studies may increasingly employ WHO cut-points, since they represent a more robust criterion-based standard, we recommend that studies use the WHO cut-points for categorizing childhood overweight/obese in SSA, as this would allow for improved comparability and time trend analyses as attempted in this paper. A repository of studies, particularly those that are representative may be set up to this end, to allow for periodic comparative analysis for the whole of SSA. Measurements on more population representative samples are also required e.g., a multi-country survey using common measurement techniques and sampling procedures would be most desirable.

## Conclusion

This systematic review provides evidence for an overweight/obesity transition in school-aged children in SSA. While the weighted averages of overweight/obesity in SSA are lower, this transition to higher proportions of overweight/obesity is similar to findings in various developed countries. The weighted average of overweight/obesity was higher in girls than in boys, and higher in those with higher SES. The review also revealed a persisting problem of underweight in the region, underpinning a double burden of risk factors. Findings of this review indicate that more nationally representative studies are needed to strengthen this field of research, and that interventions and strategies to address the growing threat of childhood overweight/obesity should focus on the higher SES and urban populations, with greater attention placed on girls.

## Acknowledgments

The authors are grateful to Alison McFarlane and Afekwo Mbonu for their contributions towards locating the full text articles and for assistance with manuscript formatting.

## Supporting Information

Checklist S1 PRISMA checklist.
(DOC)
