# Correction: Evidence of an Overweight/Obesity Transition among School-Aged Children and Youth in Sub-Saharan Africa: A Systematic Review

**DOI:** 10.1371/journal.pone.0101098

**Published:** 2014-06-19

**Authors:** The PLOS ONE Staff

Figure 1 is missing a superscripted “c”? from the box titled “Studies included in synthesis (n  =  283)”. The authors have provided a correct version of Figure 1 here.

**Figure 1:**
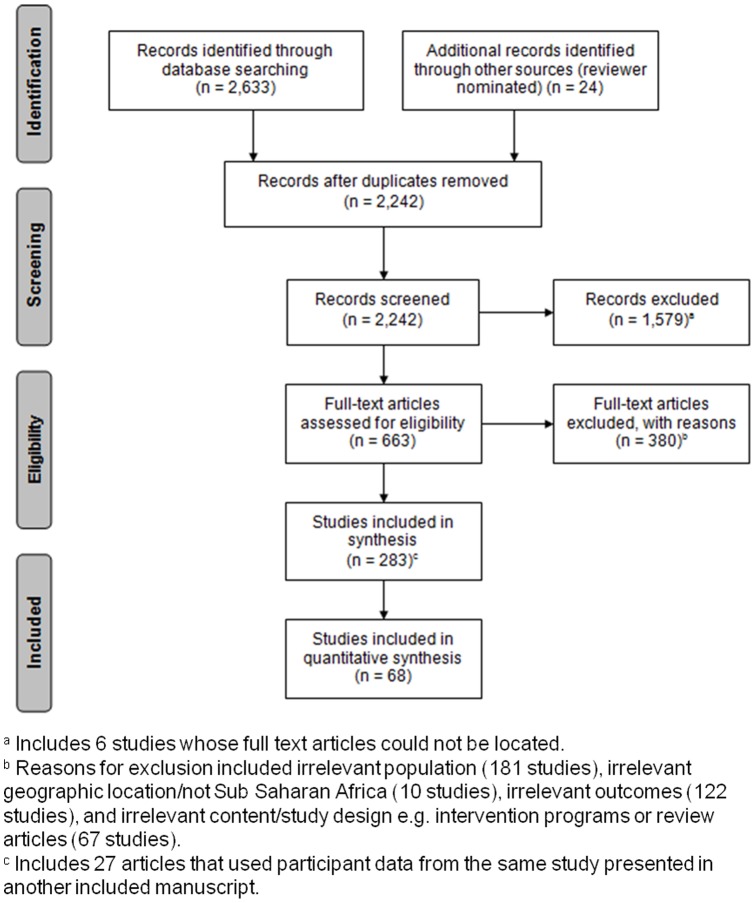
PRISMA flow chart of search strategy results.
